# Management of Patients Who Make Threats Against Elected Officials: A Case Report

**DOI:** 10.3389/fpsyt.2018.00177

**Published:** 2018-05-08

**Authors:** Paulina Riess, Luisa Gonzalez, Panagiota Korenis

**Affiliations:** Bronx-Lebanon Hospital Center, New York, NY, United States

**Keywords:** Secret Service, schizoaffective disorder, threats, elected officials, Duty to Warn

## Abstract

Federal law makes it a crime to threaten the President of the United States. The Secret Service conducts thousands of violence risk assessments each year. Literature suggests that 75% of individuals who make threats have been diagnosed with a mental illness (1). Studies show that prominent symptoms in presidential assassins include persecutory and grandiose delusions, hence falling into the category of psychotic disorders. We present a case of a patient diagnosed with Schizoaffective Disorder brought to CPEP (Comprehensive Psychiatric Emergency Program) by the Secret Service for repeatedly dialing 911 and making threats to the President. In the past year the patient had been hospitalized three times for similar behavior. Initial presentation included acute symptoms of psychosis and mania including persecutory delusions, command auditory hallucinations, grandiosity, and thought disorder. Clinicians were faced with unique challenges and consulted the forensic service to navigate the role of the Secret Service and develop a plan to prevent future episodes. The patient was discharged with a court order for treatment, long acting medication, as well as an outpatient appointment. The treatment plan has been effective and the Secret Service has ceased their investigation. We aim to explore issues in patient confidentiality, duty to both report and protect. We will also provide strategies and recommendations for such patients on the inpatient unit.

## Introduction

The Secret Service conducts thousands of violence risk assessments each year. The Secret Service has access to accurate criminal histories that enable the agency to more thoroughly investigate threats (2, 3). The authors of the cited publication state that the Secret Service recommends all manner of threats to be reported. Direct threats against public figures should never be ignored since a proportion of subjects who threaten do approach but in most cases do not carry out an attack (4, 5). Ultimately, reporting a patient to the Secret Service does encompass a breach of patient confidentiality (6). Authors of another study argue that the breach of patient confidentiality may be ethical and justifiable, even obligatory if that breach prevents harm to others (1, 7, 8). According to the US Attorney's Manual Concerning Threats Against Government Officials, of the individuals who make Presidential threats, 75% have been diagnosed with a mental illness. The role of the President in our society has led to his inclusion in the delusional system of many psychiatric patients (9). Further Studies show that prominent symptoms in presidential assassins include persecutory and grandiose delusions (4). We present a case of a patient diagnosed with Schizoaffective Disorder brought to CPEP by the Secret Service for repeatedly dialing 911 and making threats to the President. In the past year the patient had been hospitalized three times for similar behavior. Initial presentation included acute symptoms of psychosis and mania including persecutory delusions, command auditory hallucinations, grandiosity, and thought disorder. This patient's delusions encompassed ideas of reference such as beliefs of manipulation by white house officials via an inserted chip. Clinicians were faced with unique challenges and consulted the forensic service to navigate the role of the Secret Service and develop a plan to prevent future episodes. The patient was discharged with a court order for treatment, long acting medication, and an outpatient appointment. The treatment plan has been effective and the Secret Service has ceased their investigation. We aim to explore issues in patient confidentiality, duty to both report and protect. We will also provide strategies and recommendations for such patients on the inpatient unit.

## Case presentation

### Patient information has been de-identified for protection from possible negative consequences

The subject of our case study is a patient within the age range of 30-40 years, carrying a diagnosis of Schizoaffective Disorder, brought to the CPEP by the secret service for repeatedly dialing 911 and making threats against the President of the United States. The patient made delusional remarks stating that the secret service agents were involved for dialing 911 and stated that President and First Lady made derogatory remarks via a satellite chip planted in situ. The patient stated that the intention or plan of killing the president were absent. The patient demonstrated ideas of reference, grandiosity, and thought derailment. The patient refused treatment with psychotropic medication upon admission to the unit. The patient was difficult to interview and spoke in flight of ideas. According to one study, prominent symptoms in presidential assassins include persecutory and grandiose delusions (4). Authors of another study state that such patients are typically unmarried, male, paranoid schizophrenics (10). The patient was otherwise calm and cooperative throughout his admission denying suicidal/homicidal ideation.

According to the special agent overseeing the patient's case, the patient had been making incessant telephone calls to the Secret Service office, leaving rambling incoherent messages with laughing into the phone then becoming angry. When the secret service team got to the patient's apartment the door was wide open and the patient was not there. The special agent described the apartment to be in a “deplorable” state. When the patient returned, the team found this individual to have decompensated since their last encounter, with very poor hygiene smelling of body odor. The special agent also stated that prior to the patient's last admission, threats against the First Daughters were made.

The patient was first treated by a psychiatrist in college for emotional dysregulation related to relationships and was hospitalized for the first time in 2010. The patient has had multiple hospitalizations over the past 5 years, including an admission to a state facility in 2012. The patient had been admitted to our institution many times in the context of similar circumstances, had never attempted suicide, and did not report a history of mental or developmental disorders in the family. The patient admitted to smoking marijuana every 6 months. Urine toxicology was negative for illicit substances upon admission. Past medical history was significant for HIV infection with an undetectable viral load, contracted via sexual contact. Developing a therapeutic alliance with this patient was a challenge given the intense paranoia and severity of psychotic symptoms, thus much attention was given to alliance building and collaborative treatment planning. In addition, a consultation from the Department of Psychiatry High Risk Committee was conducted and their efforts aiding in developing risk mitigation strategies for the patient. The Secret Service Agents also worked alongside the treatment team with the patient's consent to develop a plan to prevent further legal action. Our patient consented to this and was familiar with the agents working on his case, appreciating the potential legal consequences of such behavior. The patient ultimately agreed to treatment and was started on a first generation high potency antipsychotic medication. The patient accepted treatment with Haldol Decanoate 100 milligrams via monthly injection, and Lithium 900 mg once daily by mouth, and was consequently discharged with a court order for psychiatric follow up. It has been almost two years since the patient's discharge from the inpatient unit and the Secret Service has since ceased their investigation. The patient has been compliant with court ordered psychiatric treatment, outpatient follow up and medication.

## Discussion

A majority of the individuals investigated by the Secret Service are categorized into three classes. Class I includes persons who have expressed overt threatening statements but have made no overt action. Class 2 is allotted to individuals who have a history of assaultive behaviors toward authority figures, and individuals in class 3 are considered dangerous and have typically been federally prosecuted (2). When working with patients who pose such a threat, the literature recommends a risk assessment be done by a senior physician. Ultimately, reporting a patient to the Secret Service does encompass a breach of patient confidentiality (2, 11–13). Literature emphasizes that although public safety remains high in priority, the patient's best interest must be taken into account and thus the entire medical chart of the patient does not need to be disclosed. When considering patient's confidentiality, our team argues that the timing of reporting the threat to the Secret Service is important to consider. For instance, someone who presents to the emergency room acutely intoxicated or in a psychotic state who makes threats against a political figure may not have such beliefs at the time of discharge. We encourage clinicians to strongly consider patient's confidentiality and ensure they consider the mental state of their patients at the time of discharge to the community.

## Recommendations for psychiatrists

In summary we would like to provide psychiatrists with some key recommendations when treating such patients on the inpatient unit. Long acting medication should be administered to ensure compliance. The first dose may be administered during the hospitalization. A court order for psychiatric treatment should be obtained. We also recommend obtaining a second opinion from a High Risk Committee, senior psychiatrist, or forensic psychiatrist. It is also important to involve the patient in their treatment and identify ways to prevent readmission. If the Secret Service is involved, the patient's consent should be obtained to involve them in treatment planning. In conclusion, the patient should be aware of the legal consequences if he continues his behavior.

We would also like to briefly address the issue of threats against famous, non-political figures. Literature has shown that the crime of stalking has attracted media attention over the past several years. However, research in this area is lacking regarding protocol and assessment of threats against celebrity victims (4).

## Conclusion

Despite many challenges with initiating treatment, the patient presented in this paper remains compliant. This can most likely be attributed to the collaborative effort of the inpatient treatment team and the secret service agents assigned to this case (Table 1). Much time on behalf of both parties was spent engaging the patient in supportive psychotherapy and thus creating a strong therapeutic bond prior to initiating treatment. A sense of trust between the patient and clinicians had to be established in order to convince this individual to agree to long acting injectable medication as well as being court mandated to treatment. The outcome of this case has been and continues to be a success. We have kept clinical tabs on the patient via communication with the outpatient psychiatrist. The patient has remained compliant with outpatient follow up and medication. We hope that our experience with this case proves to be beneficial for other psychiatrists as they navigate their way in management of such patients.

**Table 1:** Recommendations for psychiatrists when working with patients who make threats against political figures.

**Recommendations (you should use the chart from your poster it had many more recommendations)**
Long acting depot medication
Court order for psychiatric treatment
Identify risk mitigation strategies to prevent readmission
High Risk/Second Opinion Evaluation from senior psychiatrists/forensic psychiatrist
Involve the patient and empower the patient to identify ways to prevent readmission and prevent further legal ramifications
If the Secret Service is involved, with the consent of the patient involve them in the treatment planning
Ensure that patient is aware of potential legal consequences if they continue their behavior

## Author contributions

Acquisition of data: PR. Analysis and interpretation of data: PR, LG, and PK. Drafting of manuscript: PR. Critical revision: LG and PK.

### Conflict of interest statement

The authors declare that the research was conducted in the absence of any commercial or financial relationships that could be construed as a potential conflict of interest.
